# An intelligent cross-border transaction system based on consortium blockchain: A case study in Shenzhen, China

**DOI:** 10.1371/journal.pone.0252489

**Published:** 2021-06-09

**Authors:** Zhengtang Fu, Peiwu Dong, Siyao Li, Yanbing Ju

**Affiliations:** 1 School of Management and Economics, Beijing Institute of Technology, Beijing, China; 2 Department of finance, Longfor Properties Co.Ltd., Beijing, China; University of Pisa, ITALY

## Abstract

Cross-border transactions have been more and more popular around the world. However, the current cross-border transactions still have risks and challenges, e.g., differences in regulation policies and unbalanced profits of banks. To address this critical issue, we construct a new framework for the transaction system with the support of blockchain technology. In this paper, we propose a new consortium blockchain system, namely asymmetric consortium blockchain (ACB), to ensure the implementation of cross-border transactions. Different from traditional consortium blockchain, the new blockchain system could support the supernode to regulate all the transactions timely. Furthermore, the new smart contract is designed to lower the opportunity loss for each node and make the profits allocation system fairer. In the end, the numerical experiments were carried out based on the transactions of Shenzhen and Hong Kong. The results show that the proposed ACB system is efficient to make the profit allocation fairer for the participants and keep intelligent for the new cross-border transaction system.

## 1. Introduction

With the boom of economic globalization, the cross-border transaction has played an important role in our daily life. Several main issues need to be addressed in this international trading scenario, including currency exchange, government regulation, and transaction safety, etc. [1,2]. Although, there are some emerging third-party transaction tools (e.g., Alipay, Wechat-payment), the main issues such as government regulation and trustable data sharing are still unsolved. This traditional cross-border transaction framework is hindering the process of business international and lead to much time wastes [3]. To be more specific, the transaction between A and B in a different country would have two main steps. First, the business flow should be negotiated well to make the bargain deal. Second, the cash flow should occur between the two nodes. However, the cash flow would be very slow due to the sequence transaction process, including local cash transfer, international banks handle, overseas banks handle. Besides, the transaction cost of these sequence processes is also quite high. Therefore, an intelligent cross-border transaction system is urgently needed to fulfill this gap. The cross-border transaction system should be more convenient, multi-banks synergetic, and trustable. Driven by this, we should create a new cross-border transaction framework to renovate the traditional way. Due to technology development, plenty of advanced technologies are created to make transactions more convenient. Blockchain technology is an emerging technology, which has the potential to support the new cross-border transaction system [4].

Blockchain has been proposed a decade ago by Nakamoto in 2008 [5], the initial function of blockchain is to provide a fundamental structure for Bitcoin which is a kind of cryptocurrency. The main characteristics of Bitcoin are peer-to-peer transactions and decentralized relying on consensus (e.g., Proof of work). For the merit of the blockchain such as decentralization and tamper-resistant, it is widely employed in various scenarios including financial technology, government voting management, solid waste management, and supply chain retrospect [6–8]. Blockchain has a great impact on various industries, and the financial field is the most popular field which has many innovations such as digital currency, financial assets management, decentralized ledger management [9]. The future of blockchain technology is bright and promising. It will benefit the daily life of people by providing more convenient payment services and privacy protection. Due to the trustable accounting mechanism and profit allocation, the blockchain has been regarded as a promising method to build a sustainable financial system [10,11].

To make the cross-border transaction system smarter and easily regulated, blockchain technology is introduced in the transaction system. Each block can record a part of transaction information, including payer address, payee address, transaction value, and timestamp. All these blocks are linked into a chain, which bases on the previous block hash value. Once the transactions are packaged into the specific block, the block will generate a hash value, which could ensure the data integrity in the block. The blockchain saves the whole digital currency data information, and each node could access the blockchain and get the information that they want. For example, consumer A pays the bill by official digital currency to retailer B, retailer B can access the blockchain to check the consumer’s account deposit, then compared it with the bill’s value. If the consumer’s account deposit is lower than the amount of the bill, the retailer can refuse the transaction. On the contrary, if the transaction is valid, then the transaction will be packed into a block by the node which gets the right to keep the ledger.

Due to the security and convenience consideration, the consortium blockchain system is more suitable for this cross-border transaction system. The typical characteristic of a consortium blockchain is that only partial authorized nodes can engage in blockchain maintenance (e.g., transaction accounting). Considering the regulation of official digital currency, the authorized nodes contain the government regulator (e.g., Central bank) and business bank. In traditional consortium blockchain, the selected accounting nodes have the right and duty to work together (i.e., finish accounting operation in turn). However, the role between government regulators and the business bank is asymmetric. Business banks should obey the rules, which are set by government regulators. Besides, the business bank is profit-oriented, but the government regulator is an administrative organization, which is non-profit-oriented. As a result, each business bank is willing to undertake more accounting work, so that the company can earn more transaction fees for profits. On the contrary, the government regulators will not engage in accounting but check all the information on the blockchain. So, the asymmetrical relationship between different accounting nodes should be fully considered.

The contribution of this paper is fourfold.

1. A novel cross-border transaction system is proposed supported by asymmetric consortium blockchain framework.
2. We proposed a new profit allocation mechanism considering the opportunity loss of each node in this blockchain system.
3. Real-time government regulation is considered to control suspicious transactions.
4. We implement numerical experiments to verify the effect of the new cross-border transaction system in the business scenario of Shenzhen and Hongkong.

The remainders of this paper are organized as follows. In Section 2, we give the introduction of the related works. In Section 3, we put forward a new cross-border transaction structure based on blockchain technology. In Section 4, we show the details of system implementation. In Section 5, we carry out the computer simulation with the cross-border transaction cases of Shenzhen and Hongkong. In Section 6, we put forward several managerial insights derive from this work. In Section 7, we give the discussion of this paper and propose future work.

## 2. Literature review

### 2.1 Cross-border financial service

The cross-board payment/transaction is widespread in the aspect of international trade, business flow, and capital exchange. In the early time, cross-border is used in the perspective of international investment (e.g., cross-bored corporation merge and acquisition). Home country banks and international banks provide payment, liquidity, and financial risk control service to assist the corporation to finish the acquisition business [12]. With the process of globalization, commodities and goods can be traded freely in the international market. The portion of this type of transaction (e.g., industry products trade, woods products trade, special local foods trade, etc.) is booming. Hence, convenient and digital financial services are highly required to support the running of business [13].

Besides, cross-border online shopping is also required a huge amount of financial support. With the effect of COVID-19, plenty of people choose e-commerce platforms to shop rather than offline merchants. The rapidly increasing online bills need convenient currency clearing and settlement systems [14]. However, the traditional cross-border payment service is quite complex, which refers to many banks and intermediary financial agents. The delay time of cash flow is always high, which hinders the development of cross-border business. Hence, the traditional cross-border financial system should be updated with the support of new information technologies. Although many third-party payment companies spring up, the high transaction fee and data privacy still need to address [15]. The blockchain-based cross-border payment system is popular in recent years, which has been adopted in cross-border payment with Bitcoin. Hence, the blockchain is regarded as a promising way to handle the aforementioned cross-border payment troubles.

### 2.2 Blockchain-based financial service

Blockchain is an emerging information technology, which integrates peer-to-peer networks, encryption algorithms, and decentralized storage [16]. Due to the security and cooperation of blockchain network, financial service is regarded as the best application scenario for this new technology [17]. There are three types of blockchain, which would be suitable for different scenarios. The public blockchain is widely and firstly used in crypto-currency such as Bitcoin, Litecoin, etc. [18]. Everyone could link into the public chain and transfer digital currency peer to peer. Hence, the public chain is the mainstream method for international digital currency release and maintenance. However, Public chain is not convenient for government regulation and has the potential for money laundering and terrorism finance [19]. On the contrary to the public blockchain, the privacy chain is adopted in intracompany finance, such as firm’ accounting, digital asset regulation. Highly privacy protection is a characteristic of this blockchain, i.e., all the nodes should be authorized before entering this system [20]. The consortium blockchain is an integration of public chain and private chain, which is regarded as the basic framework for Central Banks Digital currency (CBDC). The transaction records could be regulated by the government, and the peer-to-peer payment function would be opening for the general person [21]. Hence, the consortium blockchain deserves to be further researched.

With the development of the Blockchain, smart contract, as one of the most important components for blockchain application, is widely concerned by academic researchers and industrial technicians. Smart contracts could enable blockchain-based finance more intelligent [22]. Ethereum is a well-known blockchain that supports various smart contracts funded by Vitalik Buterin [23]. Smart contracts are a container of the code which can describe the real-world contractual agreements (e.g., transaction contract, payment protocol, etc.) and automatic accomplishment in cyber-space. Smart contracts have a great impact on the traditional field, such as financial transaction management, Law and economics, Supply chain management, etc. In the financial field, the traceability of loans is big trouble for lenders. Blockchain-based loans could ensure the loan transaction recorded supported by the automatic programmed smart contract [24]. In the domain of legal and law, the application of smart contracts can create a peer to peer trust in social with programed code. Compared with the traditional contract law and relational contracts, the smart contract is more efficient and convenient to facilitate the deal [25]. Smart contact can also be applied in the supply chain management field via creating a shared and transparent information ledger. This ledger not only facilitates the flow of goods information but also enhances a business network for multi-lateral collaboration among supply chain members [26]. Hence, smart contracts have become one of the most promising technologies in future business. The merit of the smart contracts (e.g., automatic implementation, data transparent) would remodel society and make our life more convenient [27]. Especially in the domain of financial technology (Fintech), Smart contract is supposed to renovate the traditional payment process.

### 2.3 Distributed ledger technology

Blockchain is a particular type of Distributed Ledger Technology (DLT), which is a complex information system. DLT derives from a distributed database, which is well researched by scholars and engineers [28]. DLT is maintained by the system participators, who obey the consensus such as data uploading, encryption, and decoding. All the nodes work together to maintain the system so that the central controller is replaced by these dispersive nodes. There are two layers of DLT, one is the fabric layer, the other is the decentralized application layer. The fabric layer is the IT basics and the application layer caters to system users [29]. The impressive merit of distributed leger is trustable data sharing supported by the encryption process, which can be adopted by all the nodes. Different nodes may have different functions and roles in the networks with the same data. The other critical merit of distributed ledger is concurrent access, search, and storage (i.e., many users could get their transaction feedback instantly). Due to the different goals and motivations of different nodes, the trust setting is still a problem in the distributed ledger [30].

**Fig 1:**
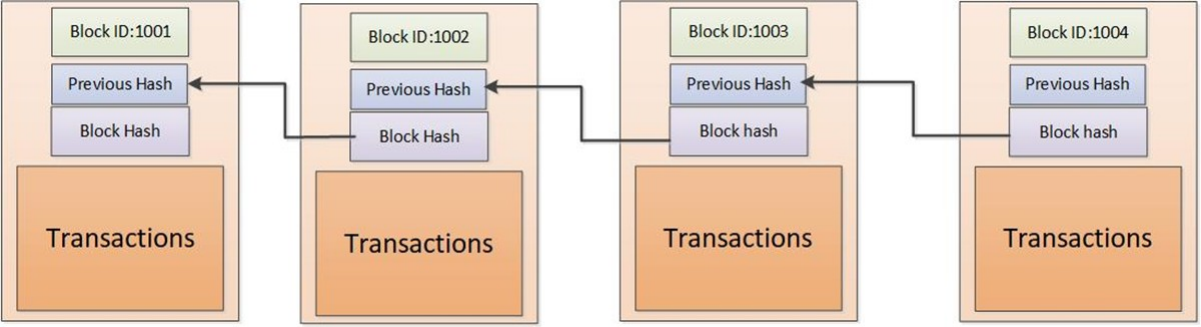
Block generating process.

There are many advanced encryption tools (e.g., hash algorithm, public, and private keys, digital signature, etc.) in DLT to ensure the trust of the system (Fig 1). The transaction information can be encrypted by public keys, and be verified via the private keys. Moreover, the transaction history can be saved permanently, even if one node defected or lost in the distributed ledger network [31]. Because of the improvement of distributed ledger and blockchain technology (e.g., more reliable, more stable, etc.), the distributed ledger is gradually employed to govern the data and undertake the job of transaction information sharing [32].

Based on the aforementioned introduction, we could know that the current research of cross-border transactions is fruitful and detailed. However, the emerging of new technology such as blockchain is not fully applied in this field, which could have a great impact on the field. Hence, this paper would focus on the combination of cross-border transactions and blockchain technology to design a novel and smart cross-border transaction system to make people’s life more convenient.

## 3 Overview of asymmetric consortium blockchain

In this section, we introduce the asymmetric consortium system in a cross-border transaction scenario and discuss the components of this system.

Different from a decentralized system or a centralized system, the multicenter consortium system can allow several critical nodes in the network [33]. These critical nodes play the role of an organizer and regulator to maintain the transaction network running smoothly. Considering the cross-border transaction system, each area or country may have its local business bank, which is trusted by the residents. These local business banks have the potential to be critical nodes in the transaction network. For the sake of government regulation, the central bank or government’s financial regulation departments also should be the critical node. Each critical node can share its information in real-time via the distributed ledger technology, and each general node should build the connection with the critical nodes as well as submitting data to critical nodes via smart contract technology [34], which derives from Bitcoin and blockchain technology [35].

However, this multicenter structure may not perfectly fit the real transaction condition. Due to the impact of government power, the government central bank of each area is quite superior to the local business bank, and the local business bank is affected by the central bank from the financial-political aspect [36]. Consequently, we considered the disequilibrium relationship between different critical nodes, and propose a new consortium blockchain structure to analyze the cross-border transaction system with digital currency.

### 3.1 System framework

**Fig 2:**
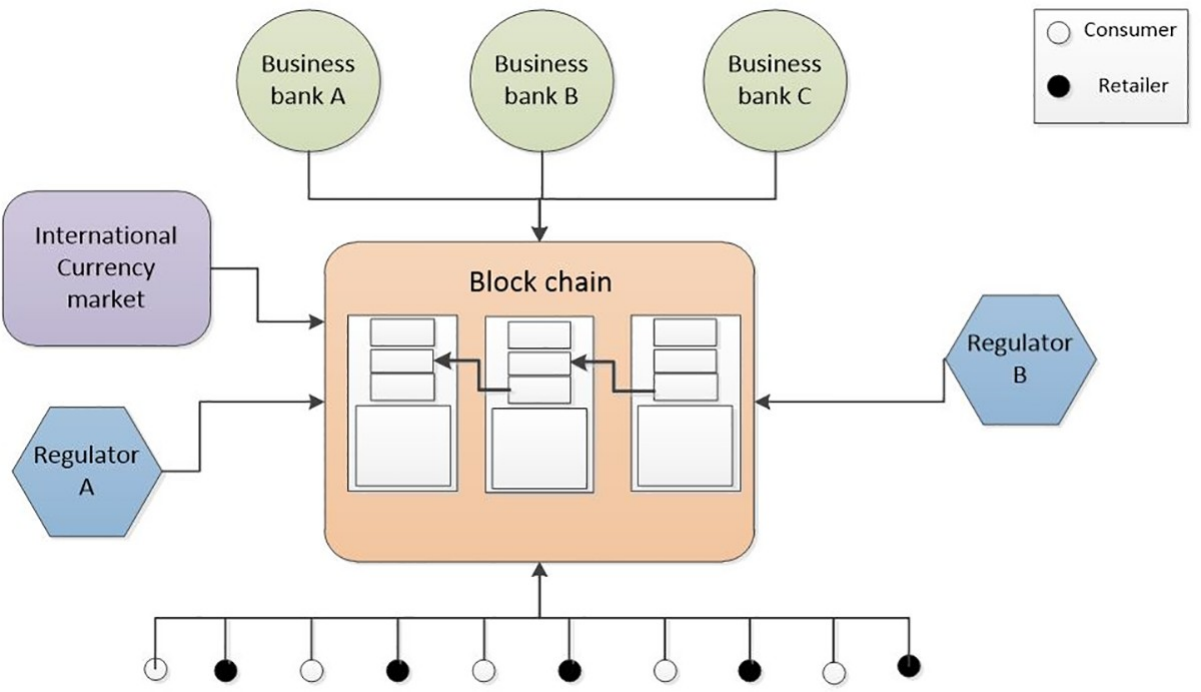
The framework of cross-border transaction.

In the asymmetric consortium blockchain system, many participants would be involved in it. The framework of cross-border transactions is shown in Fig 2 as well as the main participators in the system, including consumers, retailers, business banks, regulators, and the currency exchange market.

#### Consumer

The consumer is the initiator for the transaction from node *i* to node *j*. Each terminal device, like a smartphone, is a light node in the blockchain. Traditionally, if the consumer wants to buy out-border goods, they need to exchange their money for foreign currency in the business bank. With the development of mobile payment, more and more customers choose mobile phone Apps to finish payment [37,38].

#### Retailer

The retailer is the provider and payee of the goods in the transaction. Each retailer is also a light node in the blockchain system. Although some foreign retailers could adopt a foreign currency to pay the bill, they prefer local currency to finish the transaction. We accept the condition that foreign retailers only adopt the local currency.

#### Business bank

The business bank is the most relevant in resident daily life. Each transaction is checked and recorded by the business banks. In the blockchain system, the business bank is a full node, which saves all the transaction information of all blocks in the chain. What is more, the business bank receives the direction and command from the regulation organization, so the relationship between a business bank and regulator is asymmetric.

Before consumers go out of the border, they shall go to the business bank and exchange some foreign currency. Of course, the business bank is profit-oriented [39], consumers should pay a little exchange fee to the business bank for providing your service.

#### Regulation organization

The regulation organization is an extensive concept, including the country’s central bank, government financial department, monetary authority [40]. In the consortium blockchain system, these regulators are all full nodes, which save all the information of the whole blocks in the chain. However, these nodes do not engage in ledger accounting in our consortium blockchain system, they just play the role of information receiver, and tackle some suspicious transactions.

In cross-border transaction scenarios, the currency exchange must be regulated by the government to prevent capital flight, especially in China [41]. Consequently, once the business bank finds the transaction which is over the limitation of personal foreign currency spending, the regulation bank has the right to recheck the transaction and freeze the private account.

#### Currency exchange market

The market of currency exchange is dynamic and real-time, following the international currency market demand and supply. In the blockchain system, the currency exchange market is regarded as the exchange rate information provider. When the light nodes and full nodes need the rate information, they can turn to the market for data acquisition.

### 3.2 The details of the cross-border transaction process

**Fig 3:**
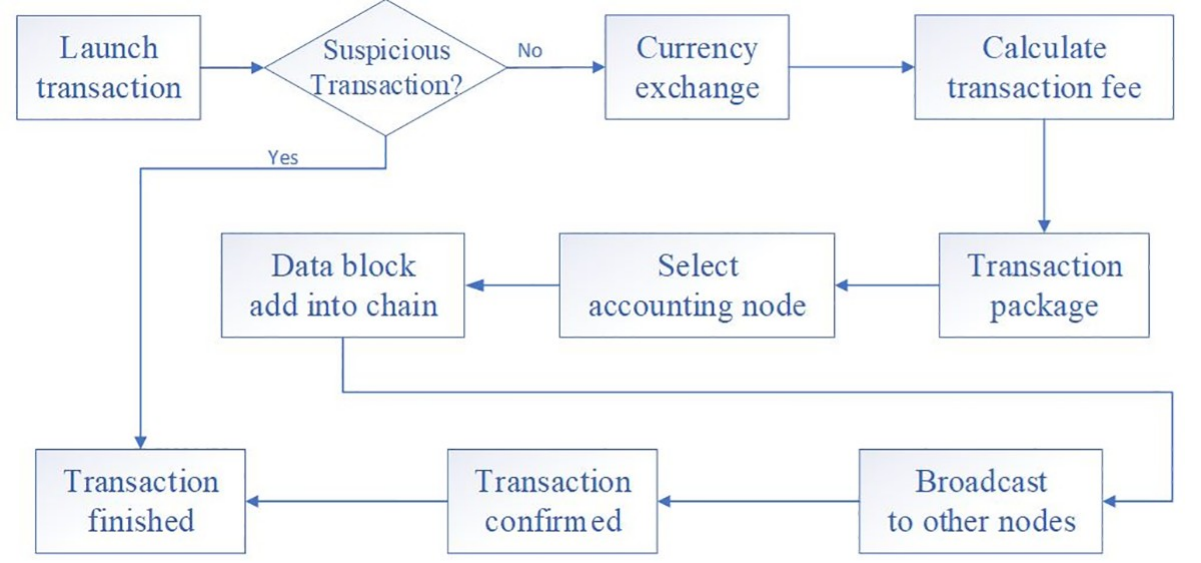
Transaction process with blockchain technology.

There are several critical steps to implement the cross-border transaction in Fig 3. From the initial transaction to the end, all the detailed information is recorded on the blockchain and well preserved. The concrete process is the following:

**Step1:** Creating the transaction. The consumer wants to buy a product in the retailer, which is out of the border. The transaction information includes the following items: consumer ID, retailer ID, transaction value, consumer belong to, retailer belongs to, timestamp.
**Step2:** Currency exchange. Due to the features of cross-border transactions, the retailer would like to accept his/her local currency. The smart is employed in the currency exchange scenario to automatically implement the currency exchange, considering the real-time exchange rate.
**Step3:** The transactions are packaged into a block, and wait to be recorded into the blockchain.
**Step4:** The business banks must check suspicious transactions. Once they seek out suspicious transactions, they should report to the regulator immediately.
**Step5:** One point gets the right to record the block into the blockchain. The selection for accounting banks follows the consensus.
**Step6:** The block adds to the blockchain following the technical regulation. The previous hash value is contained to generate the current block hash value. By doing this operation, the blockchain avoids the fork scenario, and all the transaction data in the chain is tamper-resistant [42].
**Step7:** For accounting motivation, the selected nodes have the reword (i.e., the transaction fee of the packaged block belongs to the account node).
**Step8:** After finished the previous steps, the block is chained into the blockchain. Then, the account node should broadcast the block information to the other node. The others will receive the block information, verify and accept the block as well as update the local database.
**Step9:** With the transaction finished, the consumer’ bank account subtracted the amount of currency and the retailer receives the amount of currency.

### 3.3 Model preliminary

To make the model more convenient constructed, we do the following hypothesis:

- The transaction communication system has not to delay in cross-border conditions (i.e., the delivery of transaction information from the peer to peer is instantaneous).
- In the smart contract system if the trigger condition is satisfied, the contract is dealt with immediately.
- The currency exchange rate fluctuates as time going by (e.g., the exchange rate from CNY to HKD is various each day).

Meanwhile, each node in the multicenter should obey the rule and be positive about their duty. In other words, if it is your turn to keep the ledger, you should do the job immediately. The critical nodes to keep the distributed ledger are business banks across the reference area in the cross-border transaction. What should be noticed, in this model, is that the regulator (e.g., Central bank of the government, Monetary authority, etc.) have the right to save all the transaction information, but do not engage in creating a new block as well as accounting operation.

### 3.4 List of notations

The frequently used notations are shown in Table 1, which would be involve in the following expression.

**Table 1:** Frequently used notations.

Notation	Description
N	The number of customers
M	The number of stores
W	The upper bound of privacy consuming amount
*Sj*	The total transaction value of block *j*
*R*_*pq*_	The exchange rate from currency p to currency q
*r*	Exchange currency fee for operation bank
*H*	Hash function
*HR*_*i*_	Merkle hash root function for block *i*
*B*	The whole blockchain
*B*_*size*_	The size of block
*B*_*header*_	Block header information
*B*_*i*_	The sequence of block *i*
*B*_*time*_	The timestamp of block created
*Bh*_*i*_	The Hash of block *i*
*T*	The maximal transaction amount of single block
w	The threshold money for government regulation
β	The transaction fee rate
*TS*	The transactions set
σ_*i*_	The longitude of transaction *i*
λ_*i*_	The latitude of transaction *i*
*t*_*ij*_	The transaction creates from *i* to *j*
*v*_*ij*_	Transaction value create from *i* to *j*
*time*_*ij*_	Transaction timestamp create from *i* to *j*
*SIG*_(*t*,*pk*)_	The signing algorithm working on transaction *t* with privacy key *pk*
*area*_*ijk*_	The transaction form *i* to *j* created in area *k*
*BB*	The set of business bank nodes
*BB*_*i*_	business bank *i*
*G*_*i*_	central bank delegation or regulation node *i*
*G*	The set of regulator nodes

### 3.5 Smart contract for profit allocation

**Fig 4:**
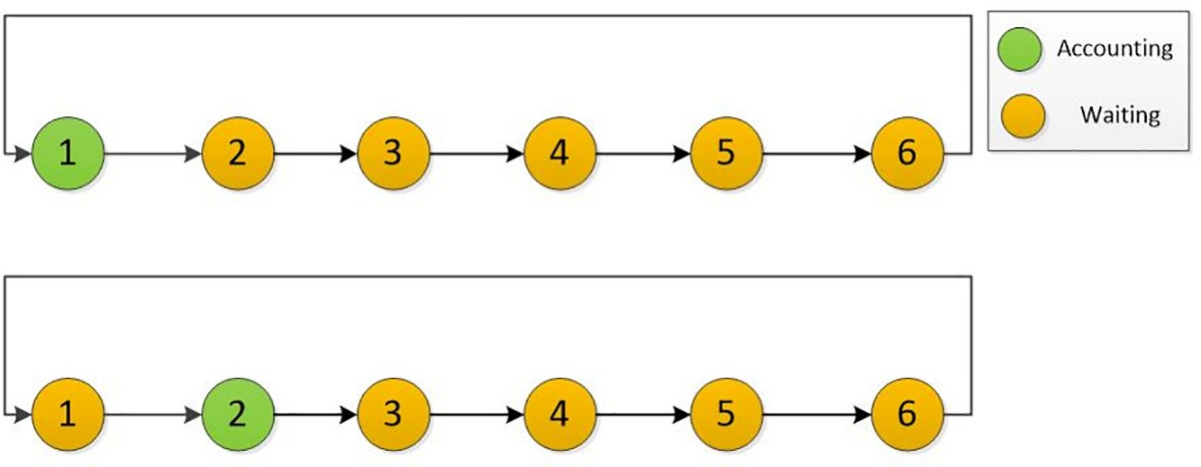
Accounting sequence.

The consensus is the regulation for blockchain, all the nodes in the network shall obey one consensus. Most population consensuses for blockchain are POW (Proof Of Work) or POS (Proof Of Stake) in Bitcoin and Ethereum. In terms of consortium blockchain, DPOS (Delegated Proof Of Stake) is widely used in this type of chain, the selected nodes are record blocks and get the transaction fee in turn (Fig 4).

However, in real business scenarios, these accounting nodes are asymmetric. The larger bank would choose to reject to engage in the blockchain considering opportunity loss. In other words, this bank could get a lower profit if they enter the blockchain system. For example, the different business bank has different attributes, such as account user number, daily transaction frequency, operation cost, etc. The transaction fee should redistribute fairly and rationally considering the different attributes. The reasons for redistributing profits are twofold.

**Fig 5:**
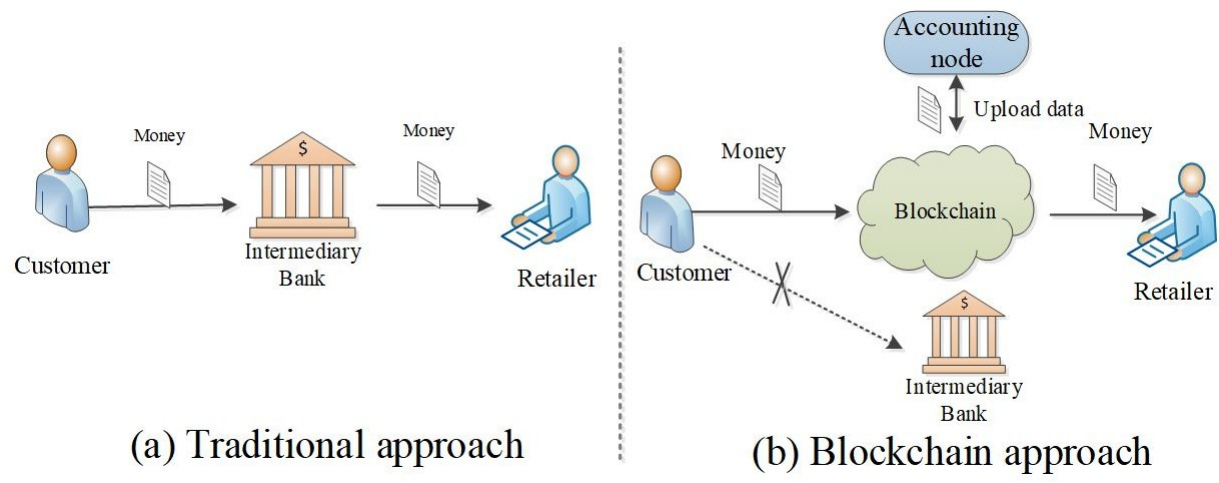
The comparison of transaction fee charging.

**Fig 6:**
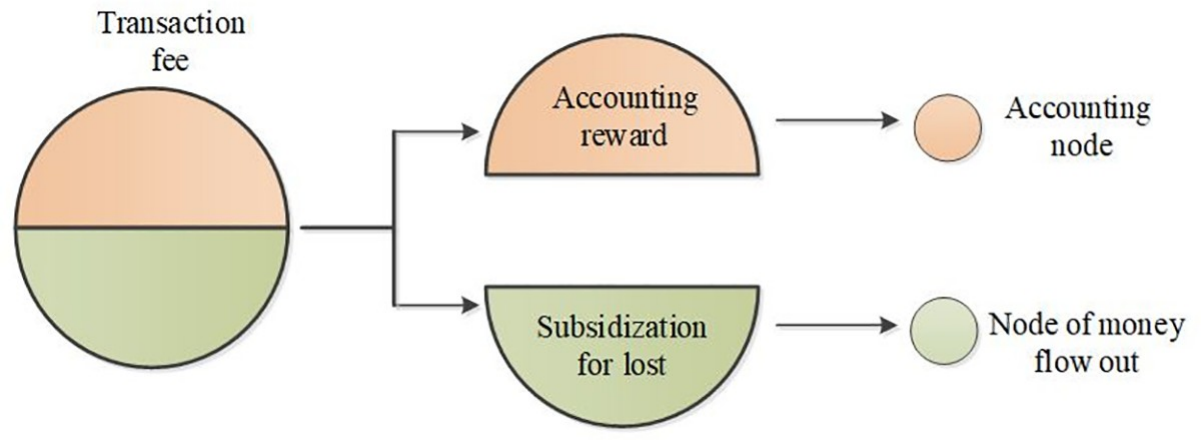
The division of transaction fee.

i) Economics consideration: The transaction frequency is a critical index, which is highly relevant to the profits of each bank. In a traditional cross-border transaction system, the transaction fee is taken by the bank, which transfers money out. However, in the blockchain system, the transaction fee is taken by the accounting nodes in turn. In other words, the large transaction scale bank would lose this portion of profit after entering the blockchain system as Fig 5 shows. So, they will refuse to engage in the system until the profits sharing mechanism changed.

ii)Technics consideration: The accounting mechanism should be adjusted to fit this asymmetric consortium blockchain system. The traditional mechanism for consortium blockchain is sequential (i.e., each supernode gets the accounting rights in turn). However, we should divide the transaction fee into two parts, one part belongs to the accounting fee for accounting nodes, the other part belongs to the bank of money out (Fig 6).

**Theorem 1.** For each block of the asymmetric blockchain system, the transaction fee can be divided into two parts as a fairer approach.

**Proof:** The profit of each node *i* is described by *π*_*i*_∈Π.The allocation model is proposed to make the profits sharing fairer in Eqs (1–4). $\pi_{i}^{1}$ is the accounting profit in Eq 2. $\pi_{i}^{2}$ is the subsidy in Eq 3. The *π*_*i*_ means the sum of accounting profit $\pi_{i}^{1}$ and subsidy $\pi_{i}^{2}$ of each bank in Eq 1. The *x*_*ij*_ only has two value (0 or 1) in Eq 4, if the conditions satisfied, the value of *x*_*ij*_ equals 1. Similarly, if the conditions are not satisfied, the value of *x*_*ij*_ equals 0. So, the transaction fee can be divided and sent to the corresponding node.


$$\pi_{i}=\pi_{i}^{1}+\pi_{i}^{2}$$ (1)

$$\pi_{i}^{1}=\rho \times \beta \times \sum_{i=1}^{n}\sum_{j=1}^{m}S_{j}\times x_{ij}$$ (2)

$$\pi_{i}^{2}=(1-\rho )\times \beta \times \sum_{j=1}^{l}v_{ij}$$ (3)

$$x_{ij}=\left\{ \begin{array}{c} 1,block\;i\;recorded\;by\;node\;j\\ 0,block\;i\;unrecorded\;by\;node\;j \end{array}\right.$$ (4)

## 4 The implementation of blockchain

### 4.1 Blockchain generation

Block generation includes several critical steps. Step1, each transaction should be verified for the validity of payment. Step2, the verified transactions should be packaged into the block *i*, and wait to be accounted for. Besides the transaction data, the block also has the block header information (e.g., Merkle tree root, block hash, etc.). Each pair of transactions can generate a hash to keep the data un-tampered. In the end, the hash value of each pair of the transaction will transform into a Merkle tree. Each Merkle tree have a root value, called hash root *Bh*_*i*_. The root value *Bh*_*i*_ is also recorded into the block header. Step3, each block *i* has a hash value, which is based on the previous block *i*−1 hash value. So, the separated blocks could link each other in a chain with the hash *H*. Step4, the accounting node will be selected by a consensus mechanism. Meanwhile, smart contracts are also activated in the process of blockchain generation. Algorithm 1 shows the blockchain generating process. Input the transaction information, including transaction timestamp *time*_*ij*_, value *value*_*ij*_, payer *i*, payee *j*, and total statistical transaction *T*.

**Algorithm 1:** Blockchain generation

**Input:***v*_*ij*_, *T*, *t*_*ij*_, *SIG*_(*t*,*pk*)_, *time*_*ij*_,

Output: an asymmetric consortium blockchain *B*

1.*p*,*BB*,*G*←∅

2. *Initial t*_*ij*_

3. *SIG*_(*t*,*pk*)_← sign transaction *t*_*ij*_ with private key *pk*

4. for account *t*_*ij*_<*T*

5. *B*_*i*_←*packaget*_*ij*_

6. end

7. *BB*← verify the valid business bank node *BB*_*i*_

8. *p*← random selected accounting node *p* from *BB*

9.*Bh*_*i*_← generate block *i* hash with hash function *H* based on previous *Bh*_*i*−1_

10. *HR*_*i*_ ← generate transaction Merkle tree root in block *i*

11. broadcast block information *B*_*i*_

12. block *B*_*i*_ saved by *G*_*i*_∈*G*

13. *G*→ verify the valid regulator node *G*_*i*_

14. Return *B*

### 4.2 Profits reallocation

The concrete implementation of for-profits reallocation is two steps. The first step is to check the accounting node income *π*. According to the transaction fee rate *β* and accounting node profit weight *ρ*, calculate the profit amount *ρ*×*β*×*S*_*j*_ for block *j*. The second step is calculating the subsidy amount (1−*ρ*)×*β*×*value*_*ij*_ for each cash flow out node *i*. With the sequence of blockchain going on, the profit is divided automatically and transfer to an accounting node and transaction relevant node. Algorithm 2 shows the process of profit allocation. The inputs are some parameter of allocation (e.g., the transaction fee rate *β*, transaction value *v*_*ij*_,etc.) and the output *π*_*i*_ is the total profit of node *i*.

**Algorithm2**: consensus for profit allocation

**Input:** *ρ*, *β*, *v*_*ij*_

**Output:** *π*_*i*_, i = 1,2,3,…,n

1 *π*_*i*_←∅

2 for *B*_*i*_∈*B*

3 Verify(*B*_*i*_)

4 $\pi_{i}\leftarrow \rho \times \beta \times \sum_{i=1}^{n}\sum_{j=1}^{m}S_{j}\times x_{ij}$

5 end

6 for *v*_*ij*_∈*TS*

7 $\pi_{i}\leftarrow \pi_{i}+(1-\rho )\times \beta \times \sum_{j=1}^{l}v_{ij}$

8 end

9 **Return** *π*_*i*_

### 4.3 Design of smart contracts

There are some new smart contracts designed in the asymmetric consortium blockchain system. One is the currency exchange contract, the other one is the suspicious transaction report contract. According to the condition variation, the smart could be triggered or not.

Similar to the offline currency exchange in the bank counter, some critical information should be known to do the deal, including the transaction time *time*_*ij*_, transaction value *v*_*ij*_, exchange rate *R*_*pq*_, etc. Considering the blockchain scenario, the digital signature *SIG*_(*t*,*pk*)_ is used to track and verify the payer information. Algorithm 3 shows the currency exchange process, contains three critical steps. Step 1, verify the transaction and fix the transaction time. Step 2, get the currency exchange rate according to the fixed transaction time. Step 3, get the exchange value and pay the bill.

**Algorithm 3**: currency exchange smart contract

**Input:***R*_*pq*_, *v*_*ij*_, *t*_*ij*_ *SIG*_(*t*,*pk*)_, *time*_*ij*_, *area*_*ijk*_, *σ*_*i*_, λ_*i*_, *r*. *BB*_*i*_

**Output:** the exchanged currency value *p*

1.*p*←∅

2. verify (*t*_*ij*_ is valid)

3. check the *pk*

4.if (σ_*i*_, λ_*i*_)∉*area*_*ijk*_

5. *R*_*pq*_←get real-time exchange rate of *time*_*ij*_

6. *p*←*v*_*ij*_×*R*_*pq*_

7. reserve *pk* in *SIG*_(*t*,*pk*)_

8.end

9.**return** *p*

Suspicious transaction recognition is a special function in the asymmetric consortium blockchain, which puts government regulation into consideration.

**Theorem 2.** The suspicious transaction *t*_*ij*_ can be frozen in the block and the other normal transaction in the same block is still valid.

**Proof:** algorithm 1 guarantees each block *i* has the unique address number *B*_*i*_ and each transaction only belongs to one consumer by signature function *SIG*_(*t*,*pk*)_. We could record the total consuming value of each person and set the maximal value via Eq (5). Due to the unique property of *SIG*_(*t*,*pk*)_, we compare the private key with the transaction signature and the consumer privacy key, if the private key is the same, the transaction should be accumulated via Eq (6) shows.


$$\sum_{i=1}^{t}v_{ij}\times \tau_{k}\leq w,k=1,2,3\ldots ,N$$ (5)

$$\tau =\left\{ \begin{array}{l} \;1,\;if\;pk\;of\;i\;equals\;pk\;of\;k\\ 0,\;else \end{array}\right.$$ (6)

The details of the suspicious report are shown in Algorithm 4, once the transaction packaged and add into the blockchain, the payer’ privacy key *pk* and transaction value *v*_*ij*_ will be recorded in the chain. With the rise of the transaction amount, the personal exchange upper limitation α could be over during a period *T*. As a result, each accounting node will check the block transaction information to find the suspicious user *i* via the privacy key *pk*.

**Algorithm 4**: suspicious transaction automatic report and freeze account

**Input:** *B*, *v*_*ij*,_ *α*, *t*_*ij*_, *SIG*_(*t*,*pk*)_, *time*_*ij*_

**Output:** suspicious-ID

1. suspicious-ID←∅

2.inital *total*←0

3. verify (*t*_*ij*_ is valid)

4.*time*_*ij*_ ← (transaction created time from *i* to j)

5. for *time*_*ij*_∈*T*

6.*total = total+v_ij_*

7. if *total>α*

8. *Check SIG_(t,pk)_*

9. record *pk*

10. suspicious-ID ← *i*

11. *Reportt_ij_*

12.*freeze* transaction account of *pk*

13. else

14. *Continue*

15. end

16. end

17. **return** suspicious-ID

## 5 Case study

### 5.1 Case overview

**Fig 7:**
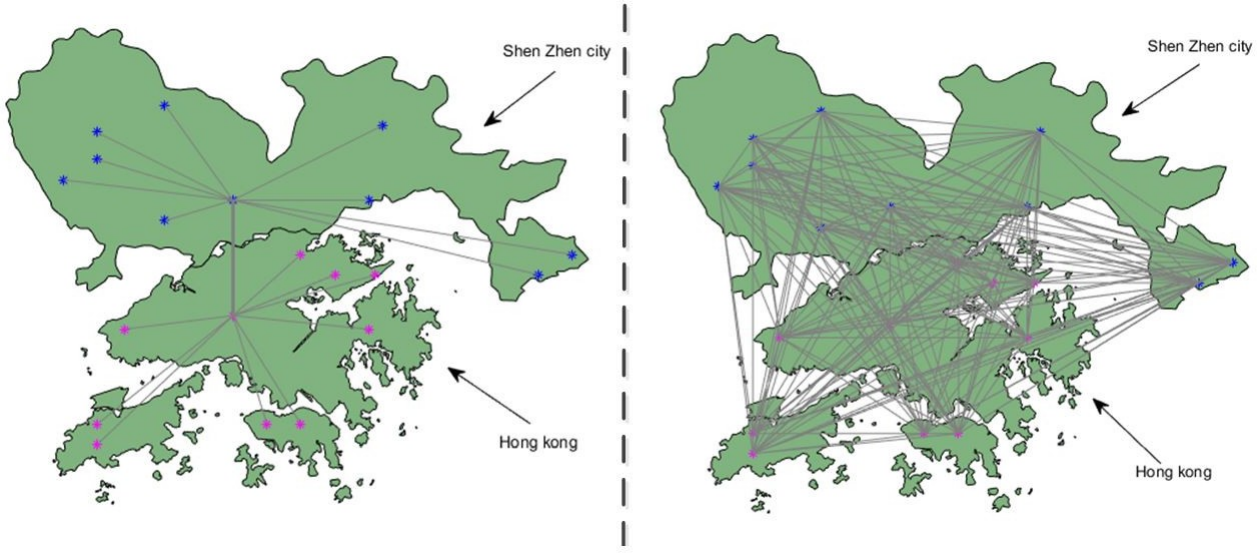
The comparison of banks information flow between Shenzhen city to Hong Kong.

Guangdong-Hong Kong-Macao Greater Bay is a huge project of the Chinses government, which aims to accelerate commercial relations. Shenzhen city of Guangzhou province is nearby Hong Kong in the Bay area, and we take up the two cities for our experiment objections. Plenty of people from Shenzhen city choose to go shopping in Hong Kong. Therefore, the requirement for safer and more convenient cross-border transactions is needed for both consumers and retailers. Traditionally, the transactions generated out of the border would have a complex process. Some small and medium-sized banks would complete the clearing process with the help of a larger bank. However, the employed of blockchain technology can solve this problem. All the banks of each area are connected and share the same ledger in the blockchain as Fig 7 shows.

Several numerical experiments are carried out to check the performance of asymmetric consortium blockchain in this scenario. We evaluate the asymmetric consortium system from two main aspects. **1)** Comparison with the traditional cross-border transaction in government regulation aspect, show the difference in the person consuming amount and bank profit in a new blockchain system. Then, do the sensitivity analysis of government regulation degree. **2)** Comparison with the traditional consortium blockchain in the profits allocation aspect, show the variation for the profit of banks, including accounting profit and subsidy. Then, do the sensitivity analysis of the allocation parameter *ρ*.

### 5.2 Preliminary works

We introduce the background parameters related to the cross-border transaction, including the government regulation degree W, transaction fee rate β, allocation rate ρ, etc. Meanwhile, the parameters for generating the blockchain are also introduced, including block size *B*_*size*_, hash function type *H* (e.g., ’MD2’,’MD5’,’SHA-1’,’SHA-256’, etc.)

**Fig 8:**
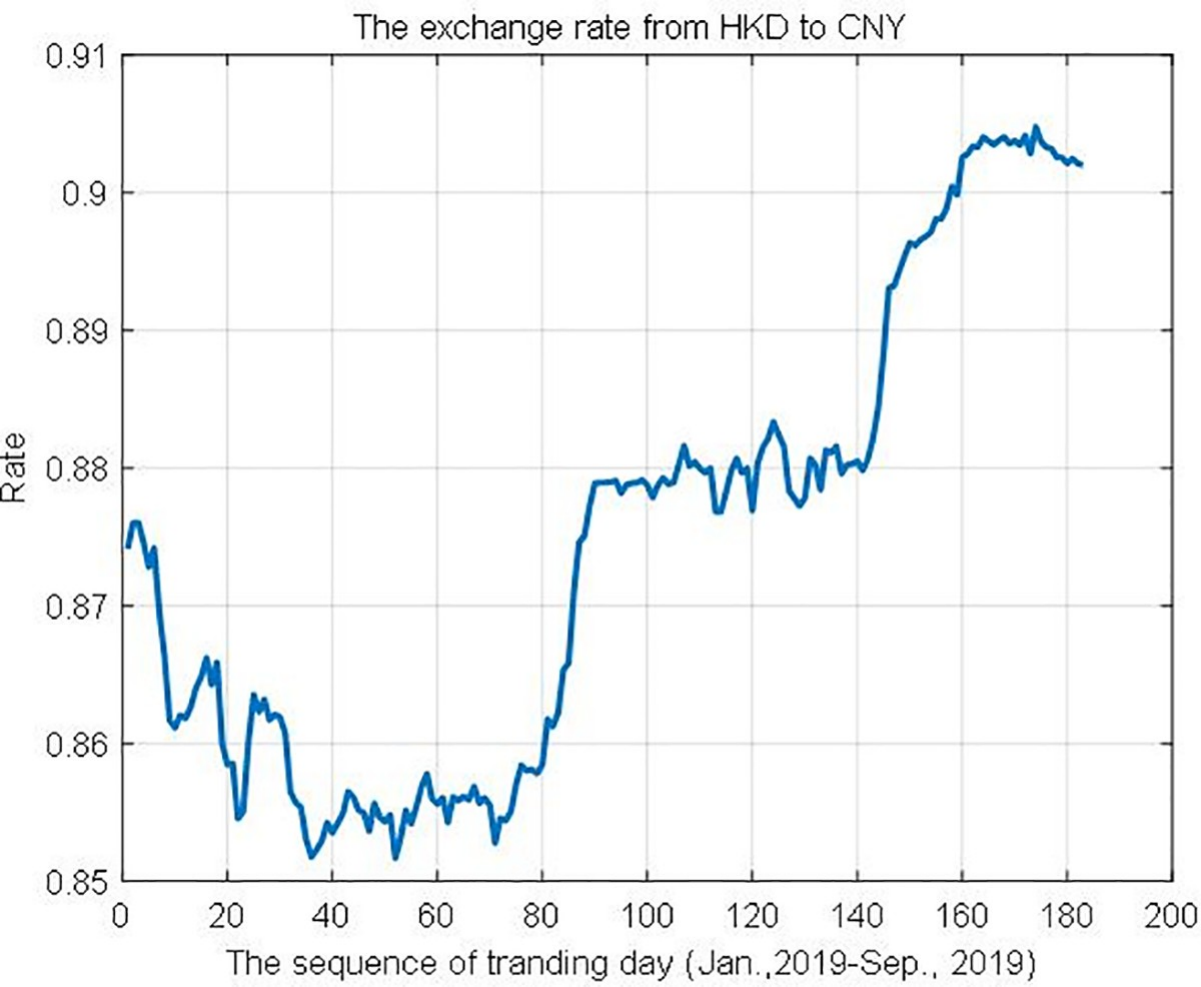
The real-time exchange rate from HKD to CNY (workday).

1. currency exchange rate and transaction data
  We collection the daily currency exchange rate between China Yuan (CNY) and Hong Kong Dollar (HKD), during the workday period from Jan. 2019 to Sep. 2019 in Fig 8. Then, we construct a data set, which has 1,000,000 transaction records, including payer ID, payee ID, transaction amount, timestamp, etc. The transactions in the dataset have the characteristic of normal allocation. The initial allocation rate ρ = 0.5, and the transaction fee rate β = 0.005.
2. Block generation parameter
  In this paper, we set the block size *B*_*size*_ = 40 and the initial hash function *H* = ‘MD5’.
3. Computer environment

The computer operating system is windows10 (64-bit) machine, Intel(R) Core(TM) i7-9750HQ CPU @ 2.60 GHz processor and 16 GB RAM.

### 5.3 Government regulation experiment

In this experiment, we introduce the proxy variable W to depict the degree of government regulation. The meaning of W is the upper bound of the person consuming amount. The initial W is set as W = 50000. If the payer consumes over W, the consumers will not be allowed to consume anymore (i.e., the later transaction of the payer is invalid automatically). As a result, the consumer personal spending amount is also influenced by the change of W. Similarly, the turnover of the retailer and the profit of banks could also be affected by the change of W.

**Fig 9:**
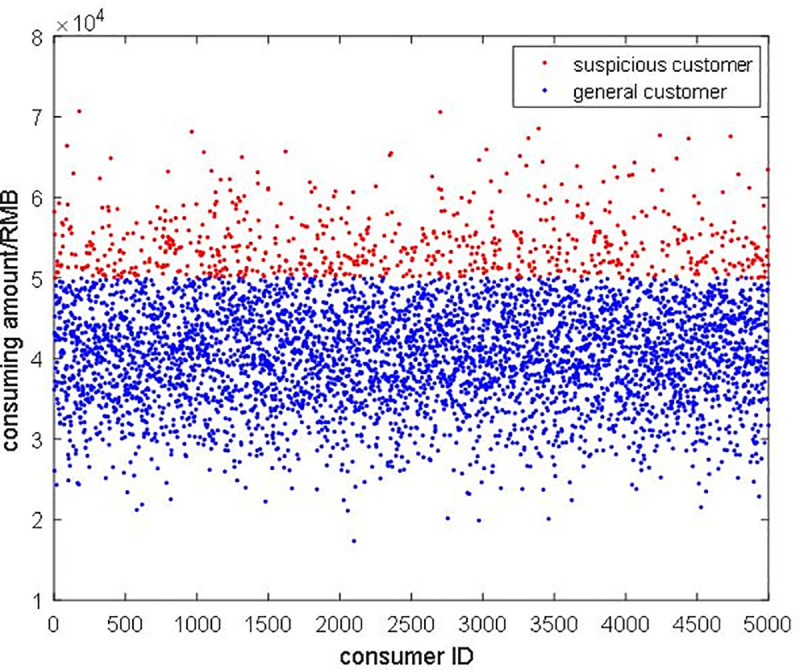
The personal consuming amount before regulation.

**Fig 10:**
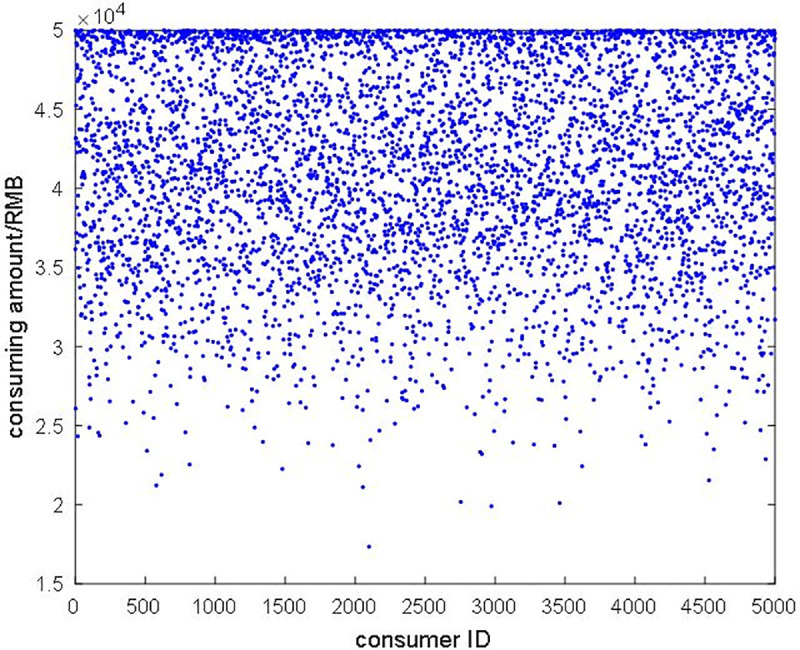
The personal consumption amount after regulation.

Firstly, we compared the traditional transaction system (i.e., the system is not regulated by the government) with the regulation system in a personal consuming aspect as Figs 9 and 10 show. The red point in Fig 9 is the consumer who consuming over government regulation degree and the blue point is a normal consumer. Fig 10 shows the regulated consuming value for consumers, and we can easily conclude that the regulation is really necessary for the supervisor.

**Fig 11:**
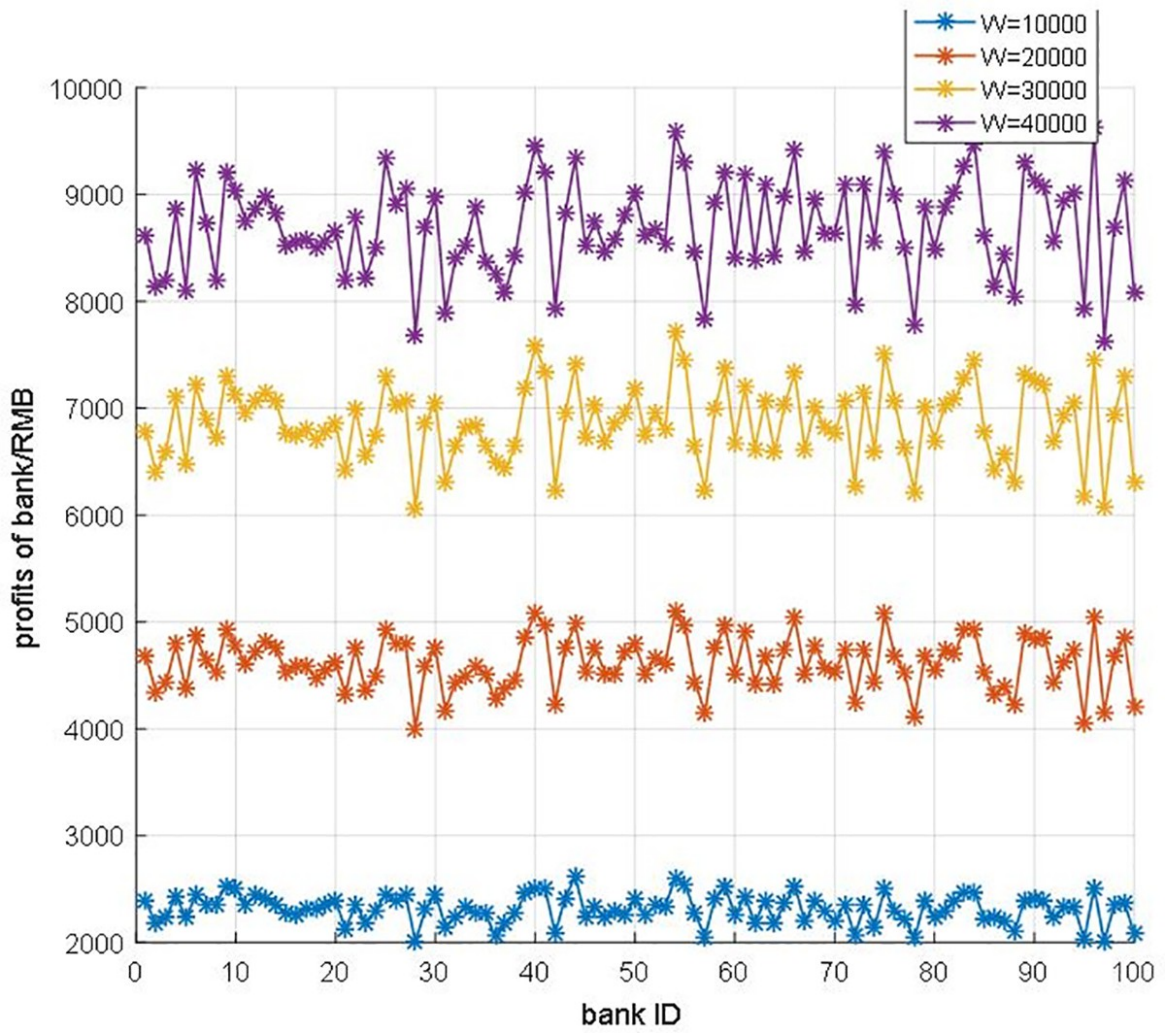
The profits of each bank in different government regulation.

**Fig 12:**
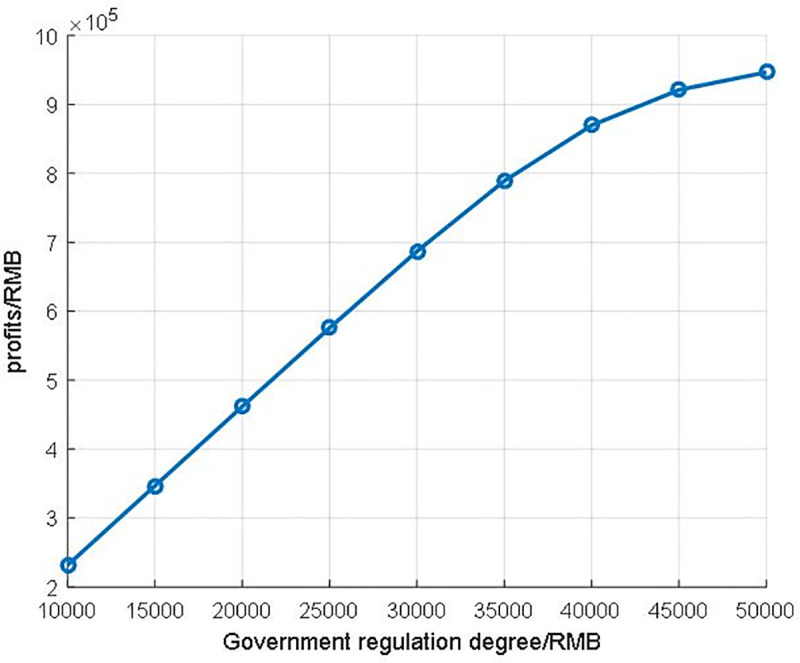
The profits of the whole bank system.

Secondly, we alter the parameter W to view the relationship between government regulation degree and profits of banks in Fig 11. The lower W means the government regulation is more severe and the profit of each bank would be lower. Furthermore, the profits of the whole bank system also affected by the government regulation degree as Fig 12 shows, and the profit of the whole bank system is showed in Table 2. As we can see from Fig 12 and Table 2, with the rise of government regulation degree, the profit of the bank system increases step by step. However, we could observe that the curve in Fig 12 is not linear, which should be concerned concretely.

**Table 2:** The banks total profits of the different regulation degree.

Regulation degree/RMB	10000	15000	20000	25000	30000	35000	40000	45000	50000
Banks total profits/RMB	231573	346714	461704	575832	686995	789090	869713	921089	946732

To unveil the relationship between regulation degree and profit of banks, we analyze the growth rate of different regulation increase gaps in Table 3. While the regulation gap raises from 10000 RMB to 15000RMB, the total growth rate of banks’ profit is 49.721%. With the strictness of regulation, the total growth rate is down sharply. While the regulation gap raises from 45000 RMB to 50000RMB, the total growth rate of banks’ profit is only 2.784%. In other words, this blockchain-based system is quite sensitive to the regulation degree, especially in the lower interval. What should be noticed is that this blockchain is a consortium blockchain, the blockchain is maintained by all the authorized nodes. Hence, the profits of all the banks are steady in the fixed regulation degree.

**Table 3:** The profit increase rate of different gap.

Regulation increase gap/ RMB	Banks total profit growth rate/%
10000–15000	49.721%
15000–20000	33.166%
20000–25000	24.719%
25000–30000	19.305%
30000–35000	14.861%
35000–40000	10.217%
40000–45000	5.907%
45000–50000	2.784%

### 5.4 Profits allocation experiment

In this experiment, we fixed the government regulation parameter W = 50000, and do a comparison with the traditional blockchain. Different allocation rates also may cause various influences on the profit of banks. Then, we carry out a test to view the relationship between the allocation rate ρ and the profit of banks. The initial allocation rate ρ equals 0.5.

**Fig 13:**
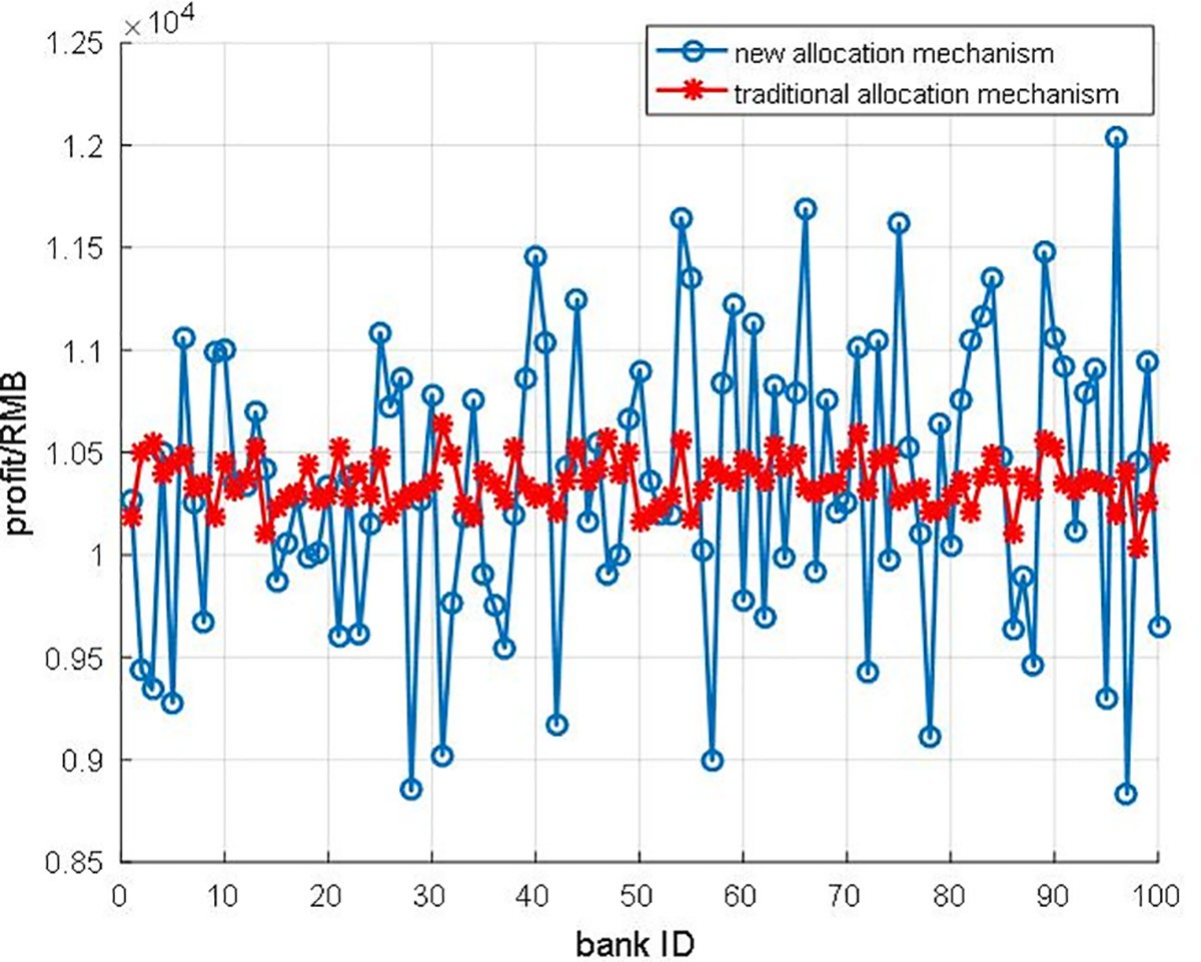
The comparison of bank profit for different allocation mechanism.

**Fig 14:**
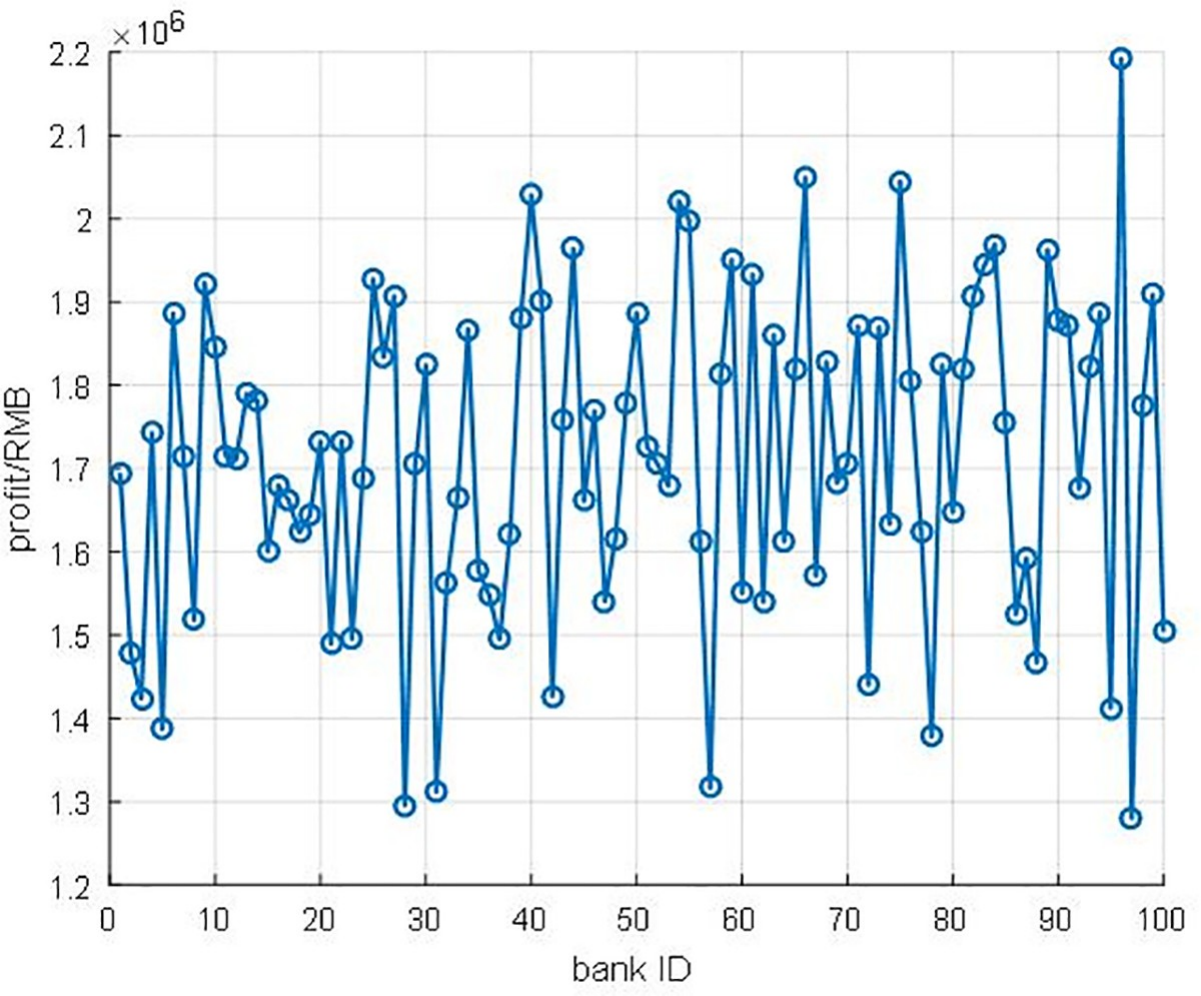
The total money out of banks.

The traditional allocation mechanism seems quite steady compared with the new allocation mechanism (Fig 13). However, considering the variation of money flow out of banks (Fig 14), the traditional allocation mechanism does not match the fluctuation for the money out of banks. We could observe that the fluctuation of the new allocation mechanism curve in Fig 13 is similar to the cash flow curve in Fig 14. Hence, we could come up with **Proposition1** as the following.

**Proposition1**: the new allocation mechanism could ensure the consistency of participants’ profits and turnover.

***Proof of Proposition 1*.** To further study the fluctuation of the bank’s profit, and the effect of the new allocation mechanism, we introduce the normalization subtract value to measurement the match rate of the allocation mechanism. There are two critical steps. Firstly, we should do the normalization operation as Eqs (7–9) shows, because the amount degree of the bank’s profit and cash flow out is different. As a result, the normalization value of new profits of each bank is x′_1_, the traditional profit of each bank is x′_2_, the money flows out is x′_3_. Secondly, we note *delta* to represent the subtract value of the different variables as Eqs (7–9) shows. The delta1 is the different value between the new profit of bank and cash flow out, and the delta2 is the different value between the traditional profit of bank and cash flow out.


$$x^{\prime }=\frac{x-x_{min}}{x_{max}-x_{min}}$$ (7)

$$delta1=|{x\prime }_{1}-{x\prime }_{3}|$$ (8)

$$delta2=|{x\prime }_{2}-{x\prime }_{3}|$$ (9)

**Fig 15:**
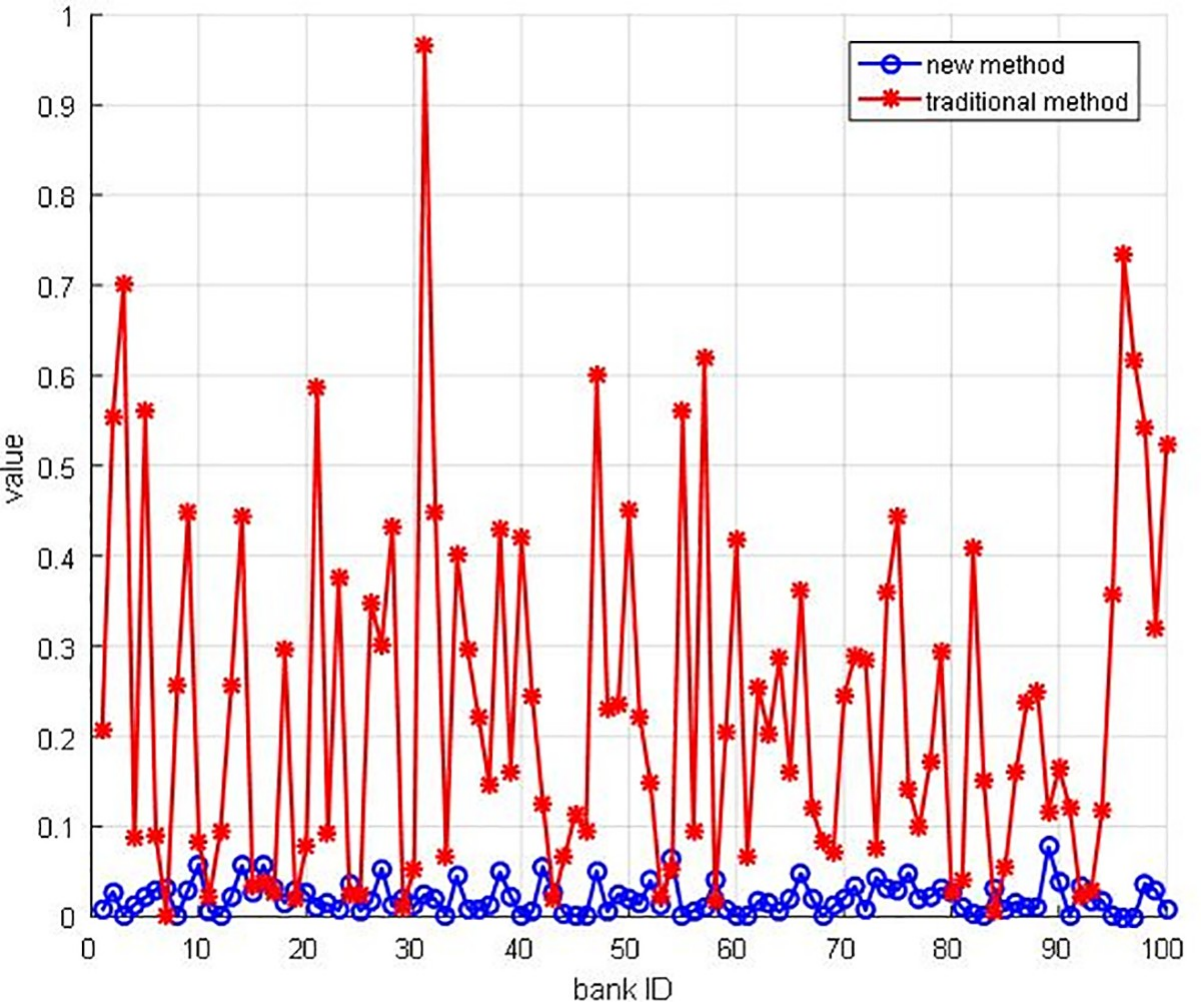
The gap of normalization value.

**Fig 16:**
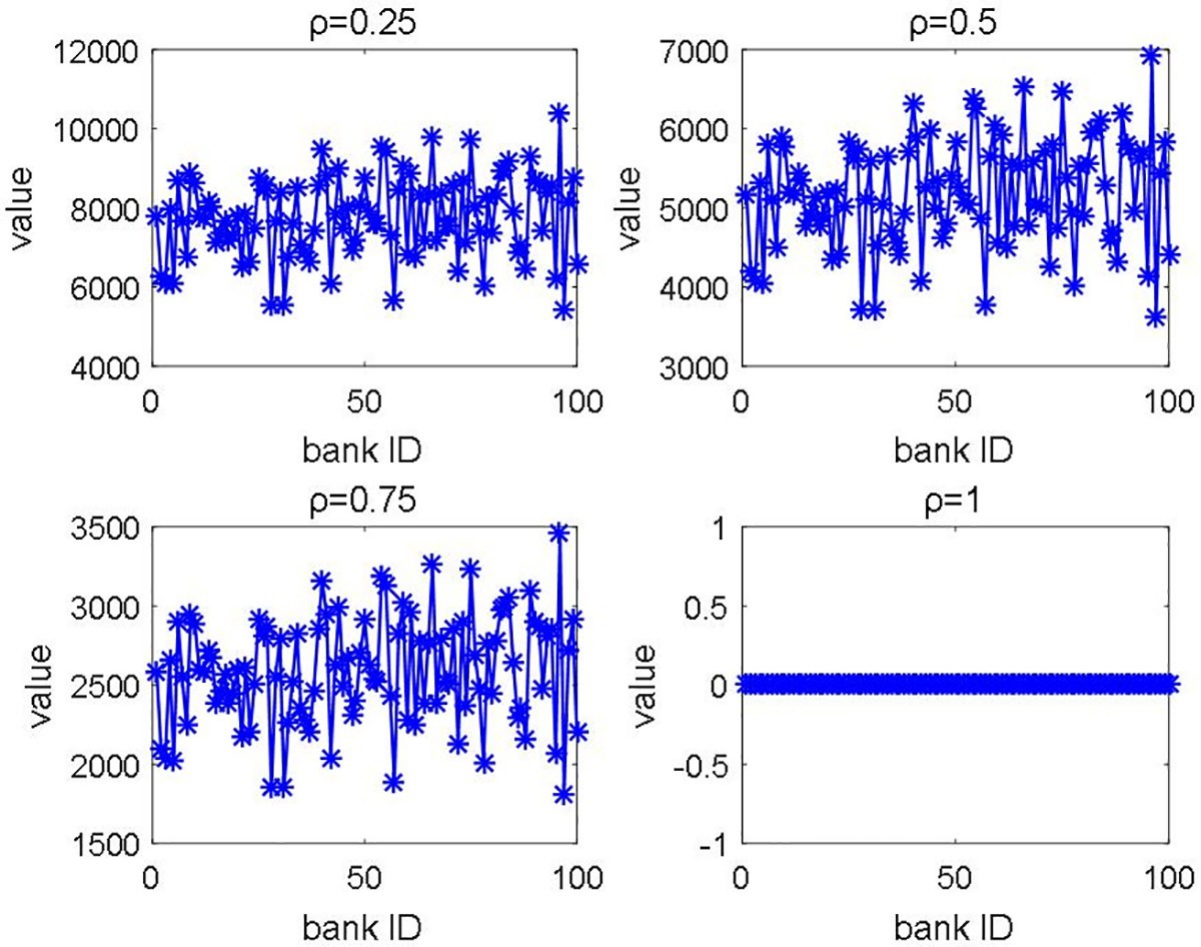
The sensitive analysis of subsidy.

The normalization results are shown in Fig 15, the line of the new method is distinctly lower than the traditional line (i.e., the new method could enhance the revenue of participants according to their capital contribution of this financial system). Furthermore, the parameter sensitivity analysis is also carried out to unveil the relationship between income and allocation rate ρ. We find out that the allocation rates ρ could lead to the differentiation of subsidy profits (Fig 16). With the increasing allocation rate ρ, the subsidy profits are reduced sharply. Supported by computational results, we have proved that the new allocation mechanism could ensure the consistency of participants’ profits and turnover. Hence, the new allocation smart contract is more practical, and more banks would be willing to engage this system.

## 6 Managerial insights

There are some helpful managerial suggestions, which could make the cross-border transaction system deployed orderly and efficiently. Meanwhile, governments could also learn some experiences and lessons from the numerical experiments.

1)The transaction value regulated by the government is critical for this system, the more rigorous regulation, the less transaction will generate. As we all know, the government always set a transaction limitation for the cross-border transaction to avoid the domestic capital flow out. However, the transaction limitation also hinders the cross-border payment as well as the commodity circulation, based on the result of the numerical experiment. Hence, we could conclude that government shall prudently set the limitation value for cross-border transaction

2) The profits allocation mechanism should be carefully designed in the business system, especially in the cooperation scenario. The profit allocation problem is critical for the sustainability of the business. In other words, we should make sure the consistency of the profits and turnover for each participant. Hence, the allocation mechanism should be well designed to incentive each participator more positive and productive. Redesigning the allocation mechanism according to their contribution is a promising way for social system.

3) Blockchain technology could be employed to ensure trust between multiple companies. As we all know, the trust relationship is very important for the business operation. However, the trust generated by people or a single company is always not accepted by all the companies. Due to the merit and framework of blockchain, this technology is especially suitable for building the trust linkage between companies, which could be regulated and managed by all participants. Besides, this “trust machine” could also build trust between companies and consumers. In this way, the blockchain could put the business process forward.

## 7 Conclusion and future work

The cross-border transaction is a complex financial system, which should be updated to catch up with the development of financial technology. In this article, we proposed an asymmetric consortium blockchain system to build a fairer and more intelligent cross-border transaction system. Government regulation is adopted to control suspicious cross-border transactions, which is rarely be concerned by previous works. Besides, the new profit allocation method is designed to ensure the interest of each node, according to their contribution to the blockchain networks. Furthermore, we carried out several simulation experiments to verify the efficiency of this asymmetric consortium blockchain system. The simulation results show that the proposed system is applicable and efficient in the real cross-border transaction scenario.

For future works, we plan to do further research on the government regulations, such as dynamic regulation, and differentiated regulation. The profit allocation mechanism should also be worth focused on to make the blockchain more intelligent and practical. Meanwhile, the impacts of new technology should also be considered in future works such as advanced currency exchange technology, digital finance, new blockchain basic frameworks, and other related technologies. These new trends could be disruptive for traditional industries and make human’ life better.

## Supporting information

S1 File (ZIP)
